# Research Progress and Model Construction for Online Health Information Seeking Behavior

**DOI:** 10.3389/frma.2021.706164

**Published:** 2022-02-11

**Authors:** Zhi-Wei Liu

**Affiliations:** Graduate Institute of Library, Information and Archival Studies, National Chengchi University, Taipei, Taiwan

**Keywords:** review, online health information, information behavior, model construction, network analysis

## Abstract

Under the background of coronavirus disease 2019 (COVID-19), online health information seeking has become one of the most important information needs of the public and even the only channel for health information seeking in this special period. A review of the research on online health information-seeking behavior will help give full play to the previous academic research, further emphasize the necessity of online health information-seeking research, and promote the development of research in this field. This study firstly presents the research overview of online health information-seeking behavior by using the informetric method. Secondly, an overview is carried out from the perspective of online health information platforms, groups, quality, satisfaction, etc., to explore the influencing factors and their relationships in the process of online health information seeking. On this basis, the existing behavioral models are integrated and sorted out to build a new behavioral theoretical model in line with the current online health information seeking.

## Introduction

Health information is generally defined as information about people's physical and mental health, illness, nutrition, and wellness. Health Information Seeking Behavior (HISB) refers to all the behaviors that people display to obtain, clarify or understand health-related knowledge, understand health risks, and prevent disease in response to specific events or situations (Manafo and Wong, 2012). Access to health information is a universal, everyday human need that will be significantly heightened in the face of a major health event in a population.

At the beginning of 2020, a sudden outbreak of the coronavirus disease 2019 (COVID-19) pandemic broke out in Wuhan and spread rapidly worldwide. Such a sudden and highly contagious global health event sent the public into a panic. Home isolation has become the main way for people to fight against this outbreak. The status of the outbreak, precautionary measures, confirmed cases, transmission routes, and health risks had become hot topics of discussion. Unlike the public health emergencies such as Severe acute respiratory syndrome coronavirus (SARS) in 2003, influenza A (H1N1) in 2009, and Middle East Respiratory Syndrome (MERS) in 2015, the background of the new coronavirus epidemic is the era of the comprehensive development of all-media and integrated media in China, which has seen unprecedented growth in information volume, communication channels, as well as information supply-side and information receiving side. The media has become more and more complex and diverse (Chen et al., 2020). According to the “Pneumonia Awareness Survey Report” released by Punch News on May 12, 67% of the public were “highly concerned” about the new pneumonia epidemic, 22% were “more concerned,” and <1% were not concerned (Li et al., 2020). In its “Epidemic Watch” report, Ariadne further pointed out that people's active information acquisition channels during the epidemic were mainly distributed on online social media platforms, comprehensive information platforms, professional news and information platforms, short video platforms, and web browsers (IResearch, 2020). The Reuters Digital News 2020 report also reveals that the outbreak of the new coronavirus epidemic has not only significantly increased the usage of online news and social media but also pushed traditional news media to a digital, mobile, and platform-driven online media environment. Nearly 65% of respondents believe that online media has played a pivotal role in helping them take the right initiatives in response to the outbreak (Fletcher et al., 2015). Overall, the sudden outbreak events have not only raised the importance of health information to the public but also highlighted that online information platforms had become the main channel for seeking health information nowadays. However, it is undeniable that while online platforms bring richer information, they are also full of distorted information and misinformation, especially excessive information that cannot be distinguished from the truth, which makes it difficult for people to find trustworthy information sources and even affects users' health information behavior and health decisions.

Therefore, a deep understanding of the characteristics of health information-seeking behavior, clarifying the influencing factors in the process of health information seeking, especially in the context of the increasingly mature and complex network environment, and reviewing and organizing previous research results will help optimize the way of publishing health information platforms, improve the quality of health information content, and enhance the efficiency of health information acquisition and utilization. It can also support government functionaries in the effective management of health information during public health events.

This study is a review of the research on online health information-seeking behavior. Online health information-seeking behavior is an important branch in the field of information behavior research in the discipline of library and information science. The important studies in this field are mainly collected in the Web of Science (WOS) database. This study uses WOS as a data source and Citespace as a text mining and visual graphical analysis tool.

## Materials and Methods

Based on the above purpose, this study firstly collates and analyzes the existing studies related to online health information-seeking behavior, and uses informetric and text analysis methods to show the overview of current research on online health information-seeking behavior; secondly, it reviews the online health information platforms, groups, quality, satisfaction, and other perspectives to discover the various influencing factors and their relationships in the online health information seeking process. On this basis, we integrate and organize them to derive a comprehensive model that meets the current online health information-seeking behavior, in order to help healthcare professionals, patients, and researchers better understand patients, their health information, communication styles, and information-seeking behavior in the future.

There have been numerous studies on online health information-seeking behavior globally. In order to cover the relevant research literature as comprehensively as possible, this study chooses the WOS database, and selected “topic” as the search field, with [TS = (“online” or “media” or “internet” OR “e-” or “digital” or “web”) AND TS = “health information” AND TS = (“seeking” or “searching”)] as the search expression, the period was unlimited, the literature type was “journal article,” and the search time was July 1, 2020, and a total of 2,036 articles were obtained. Due to the incomplete cataloging of some articles in the WOS, some records were missing and the full text was not available when searching. Therefore, through manual judgment and screening, 62 invalid articles were finally excluded, and a total of 1,974 relevant articles were retained as the basis of this study.

## Results and Discussion

### Research Progress of Online Health Information-Seeking Behavior

#### Analysis of Quantitative Characteristics

**Figure 1:**
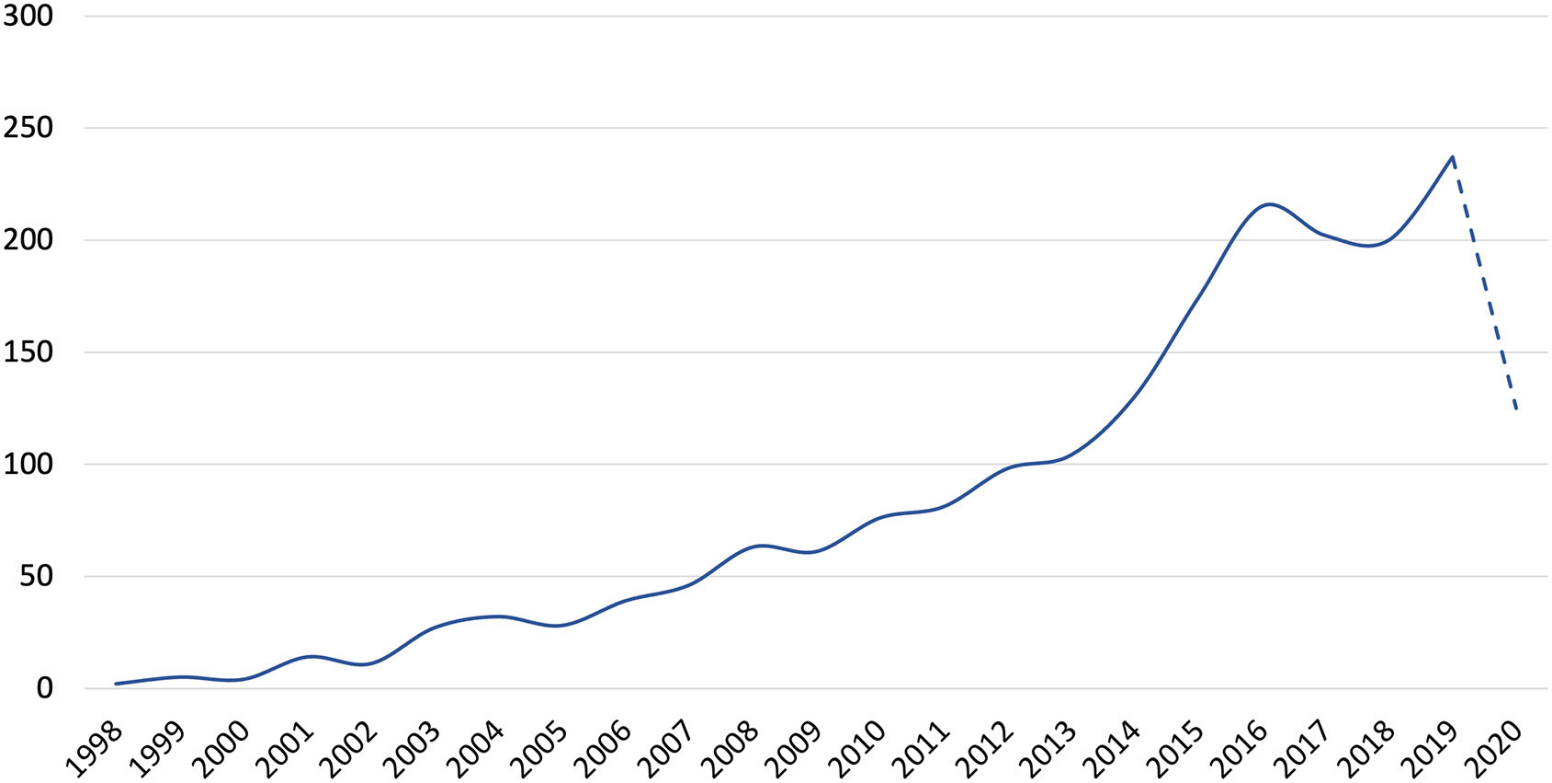
Distribution of the number of published studies related to online health information seeking behavior.

From the distribution of the number of articles published over the years (Figure 1), the research on online health information-seeking behavior began in 1988, while domestic research lagged behind and was only explored since 2009. In terms of the trend of publications, the overall growth trend of publications is stable, and the growth rate is obvious after 2013. The number of publications in 2020 (dotted line) is not yet complete, but the number of publications in 2020 is more than half of last year. The number of publications in this field is expected to keep growing in 2020.

Table 1 shows the distribution and proportion of the number of articles published in the top 10 countries according to the address of the first author of the article. From the viewpoint of the number of national publications, European and American countries are fruitful in the field of online health information-seeking behavior research. In particular, the US, UK, and Canada have more than 80% of the total number of publications. Although other countries also have some results, the gap is far from the above countries.

**Table 1:** Statistics on the number of studies on online health information-seeking behavior by country (top 10).

**Rank**	**Country**	**Number of articles**	**Percentage (%)**
1	United States	871	42.6
2	England	586	28.7
3	Canada	235	11.5
4	Ireland	68	3.3
5	Switzerland	40	2.0
6	Germany	28	1.4
7	Netherlands	28	1.4
8	Australia	16	0.8
9	India	12	0.6
10	France	9	0.4

#### Analysis of Main Research Directions

To further understand the distribution of research themes in online health information-seeking behavior, this study organized the above-mentioned 1,974 articles and converged them into the Citespace (Product by Chaomei Chen, download in https://citespace.podia.com/) tool to analyze the keyword co-occurrence status in the domestic and international research literature.

**Figure 2:**
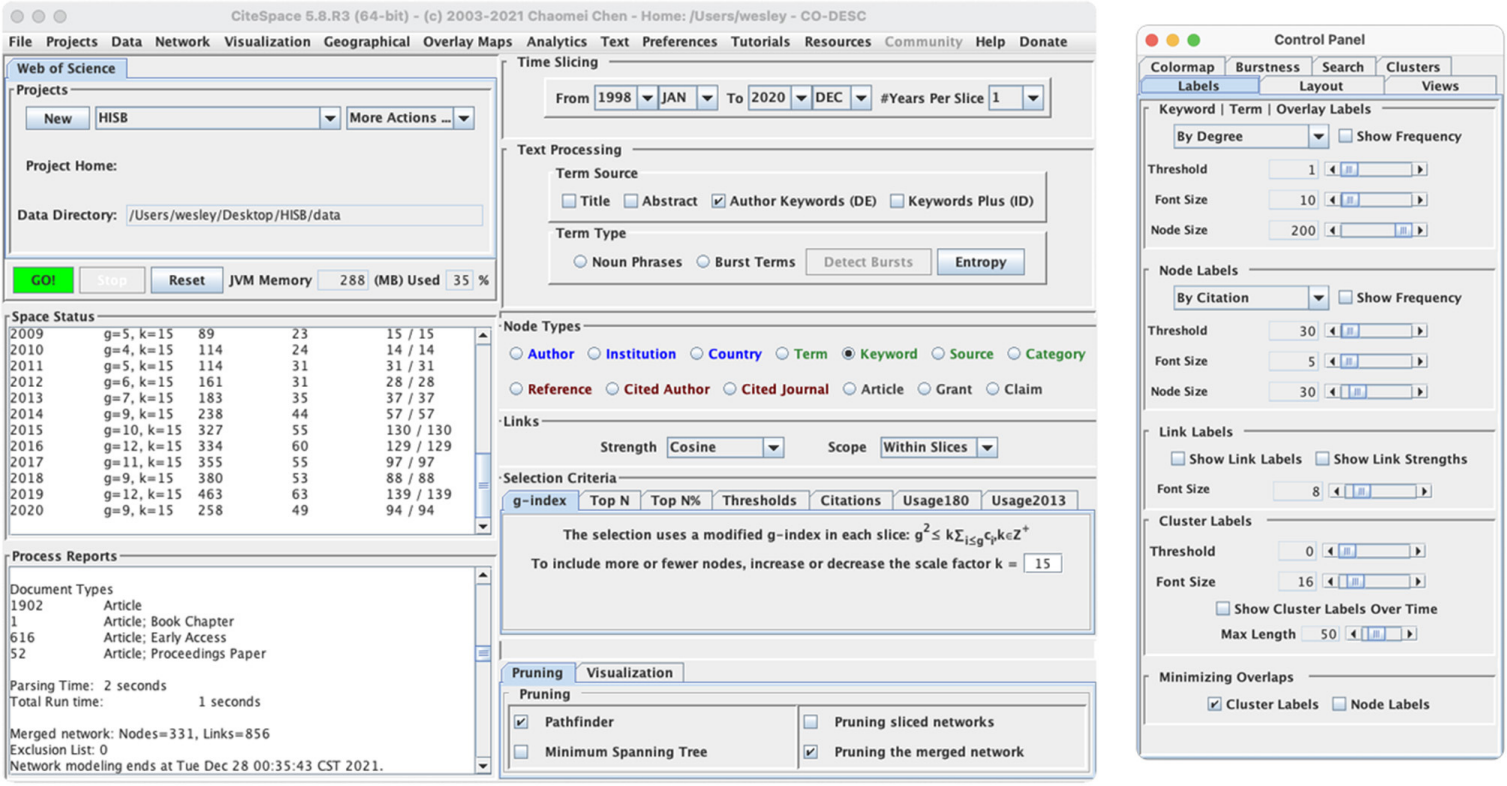
Citespace keyword co-occurrence parameter settings.

**Figure 3:**
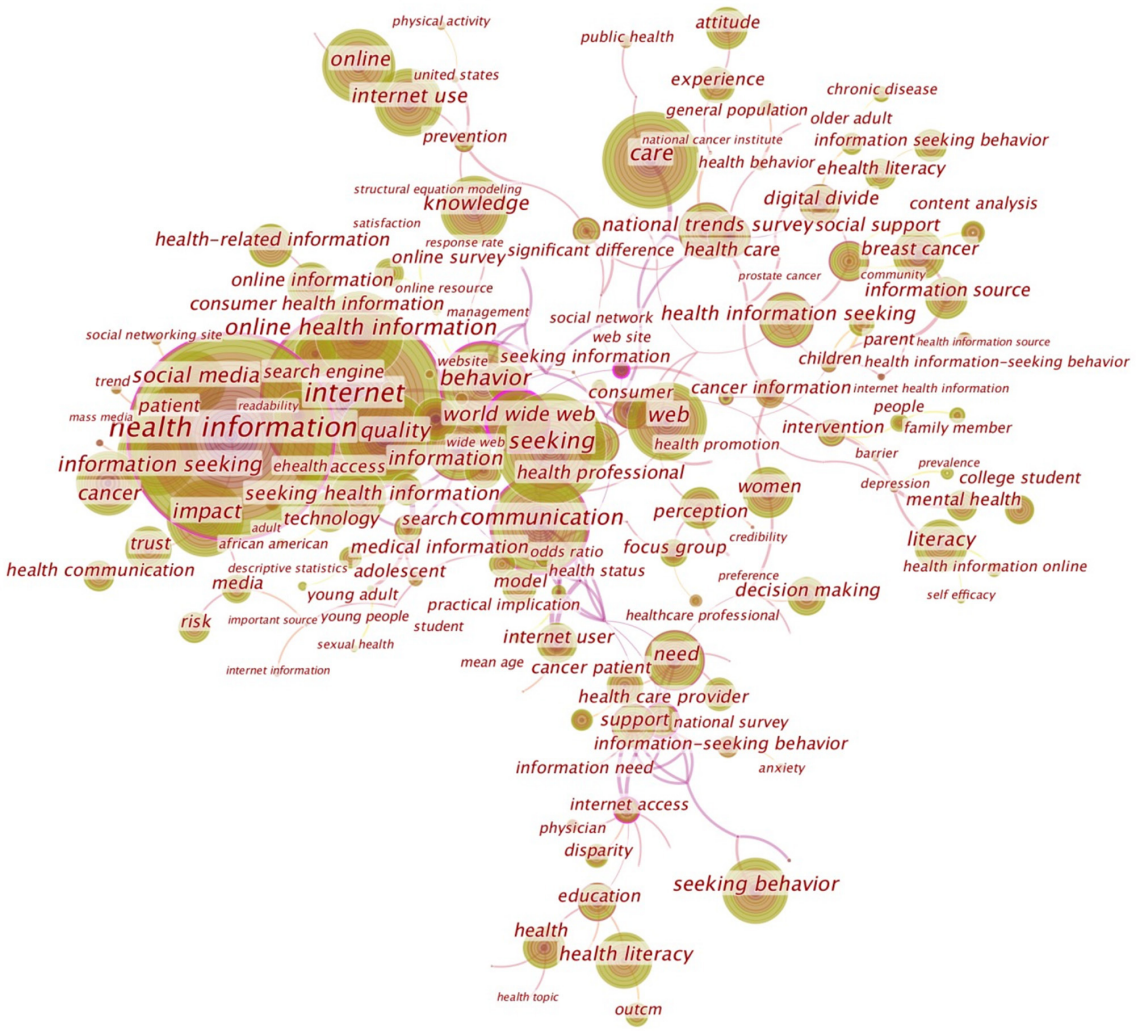
Keyword co-occurrence mapping of online health information seeking behavior research.

Keyword co-occurrence analysis is an important method used to determine research themes or research directions in a subject area. The larger the label size of the node, the more frequently the topic is co-occurred and the hotter it is; the larger the area of the node, the longer the duration of the topic; the line between the node and the node represents the co-occurrence relationship between the two nodes, and the thicker the line, the closer the relationship is. Figure 2 shows how the parameters of the Citespace tool were set. In this study, the time range was set to 1998–2020. At the same time, to cover the complete research content as much as possible and reduce the text clutter, the single time slice was set to 1 year, the text processing Term source is set as “Author Keywords (DE),” the node type is “Keywords,” the calculation criteria are set by default, and the “merged network” is pruned. Finally, the font and node size were resized according to the presentation needs. The result was shown in Figure 3.

According to the characteristics of the co-occurrence frequency of the keywords in Figure 3, this study found that, in addition to the keywords directly related to the research field such as “health information,” “information seeking,” “retrieval,” and “network,” domestic and foreign research on online health information-seeking behavior mainly focused on the following six aspects.

- Studies on information acquisition platforms, such as social media, world wide web, mass media, searching, etc.
- Studies on information-seeking populations, such as women, consumers, young adults, older adults, adolescents, and internet users, as well as status-specific ones, such as college students, nurses, doctors, patients with cancer, African Americans, health professionals, health care providers, etc.
- Studies on information-seeking content, such as mental health, prostate cancer, chronic disease, breast cancer, prevalence, sexual health, depression, etc.
- Studies on information-seeking motives, such as decision making, family member, anxiety, intervention, prevention, etc.
- Studies on information quality evaluation, such as risk, trust, impact, satisfaction, barrier, access, use, readability, quality, etc.
- Studies on influencing factors, such as knowledge, experience, gender, age, education, communication, needs, use, health status, literacy, etc.

According to the co-occurrence mapping, we can also find the common research methods in online health information-seeking behavior research, mainly focusing on content analysis, meta-analysis, focus group method, and survey method.

#### Analysis of the Evolution of Research Themes

**Figure 4:**
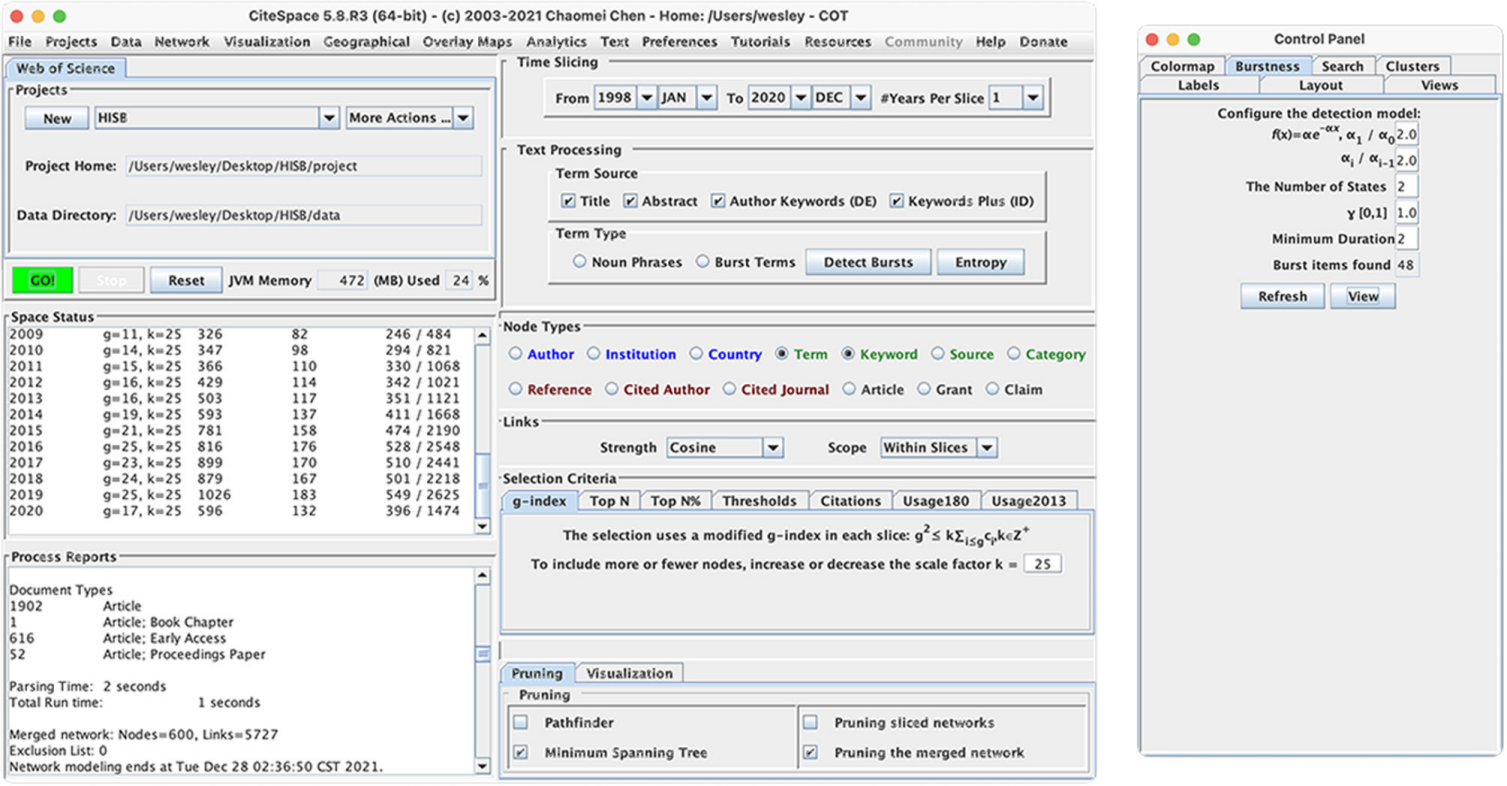
Citespace keywords burstness parameter settings.

Burstness is an important tool in Citespace for analyzing research topics and obtaining the topics' burst situation. The principle of Burstness is to analyze and extract words from the text to obtain important topic words, and to calculate the burstiness value of different topics in different periods of time, so as to identify the time area where the topic is different from the normal value and is heavily discussed and studied. Figure 4 shows the parameter settings of Burstness. The time range was set to 1998–2020, the Years Per Slice was set to 1 year. To better reflect the Burstness phenomenon, the title, abstract, author keywords, and keywords were selected as Term sources. Finally, in the control panel, the Burstness option was selected and the default parameter settings to view were used.

Table 2 uses the Citespace Burstness feature to analyze the emergence of popular topics from 1998 to 2020 using the time of keyword emergence as a cut-off dimension to describe the changing focus of researchers in the field of online health information-seeking behavior research. The long green line in Table 2 represents the entire period of the emergence of research in the field. The red area indicates the significant emergence of the related topic in the period.

**Table 2:** Keywords burstness in the study of online health information-seeking behavior.

**Keyword**	**Strength**	**Start year**	**End year**	**1998–2020**	**Theme stage**
Information tech	4.71	1998	2012	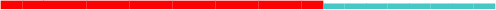	Technology Development
System	4.74	1999	2007	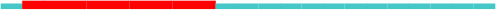	
Search engine	5.76	1999	2006	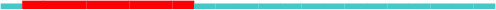	
Web site	16.53	1999	2012	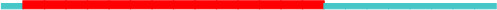	
Confidence interval	4.80	2002	2012	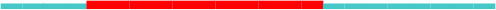	Improving the quality and awareness of information seeking
Important source	5.66	2003	2012	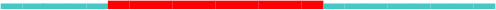	
Quality	11.47	2003	2008	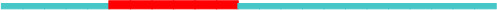	
Health education	5.02	2003	2006	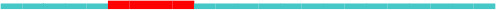	
Patient	4.31	2003	2007	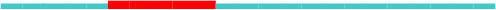	Patient-based studies
Breast cancer	3.21	2003	2011	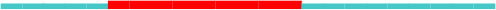	
Health service	3.63	2003	2007	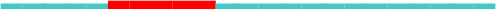	
Prostate cancer	6.07	2003	2012	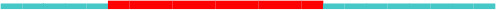	
Consumer	10.37	2004	2012	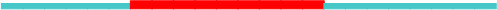	User feedback
Response rate	8.38	2004	2012	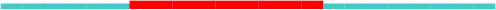	
Internet usage	4.33	2004	2007	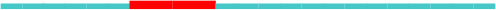	
National survey	4.99	2005	2013	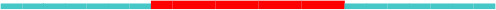	
Access	3.22	2007	2010	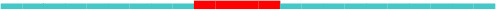	
Impact	4.01	2009	2011	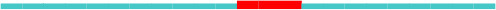	
Trend	4.88	2011	2017	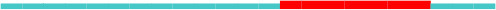	Influencing factors and future development
Barrier	5.66	2013	2016	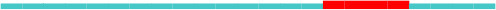	
Management	7.10	2013	2016	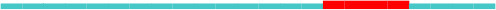	
Student	3.95	2016	2017	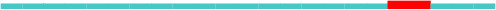	Masses-based studies
Social network	3.63	2016	2020	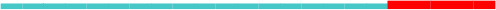	
Young adult	4.79	2016	2018	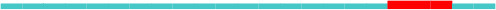	
Social media	19.71	2017	2020	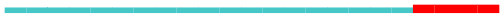	
Older adult	6.68	2017	2018	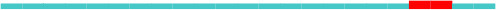	
Readability	6.76	2018	2020	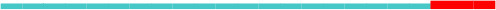	Information content assessment and demand forecasting
Self efficacy	8.03	2018	2020	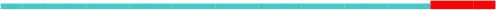	
Predictor	6.51	2018	2020	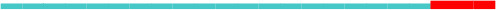	
Satisfaction	6.76	2018	2020	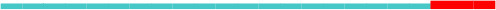	

Based on the keyword meaning characteristics and the actual literature research, this study categorizes the research themes in the field into seven main thematic evolutionary stages, which are as follows.

- The stage of technology development (1998–2012), with the implementation of information retrieval's underlying technology as the main research object, mainly exploring the construction and implementation of health information retrieval systems, retrieval tools, and retrieval platforms.
- The stage of improving information quality and seeking awareness (2002–2012), measuring and evaluating the quality, credibility, and source channels of online health information, and emphasizing health education's role to improve the awareness of online health information seeking.
- The stage of targeting disease patients (2003–2011), by focusing on exploring the online health information-seeking behavior of disease patients, especially cancer patients.
- The stage of user feedback (2004–2013). Understanding the response status, accessibility, usability, and impact of access in the online health information seeking process through survey research.
- The stage of influencing factors and future development (2011–2017), to explore the obstructive factors affecting online health-seeking, predict future development trends, and improve online health information management methods.
- The stage of research targeting the general public (2016–2020), thanks to the popularity of mobile networks and social network development, the flow of health information has penetrated social network platforms. The general public has become the main research target of health information seeking.
- The stage of information content assessment and demand prediction (2018–2020). The arrival of the all-media era has led to the diversification of health information dissemination channels and varying information quality. However, the development of information technology has provided unlimited possibilities for online health information seeking. The readability, utilization, satisfaction, and potential demand of information have become the main objects of research at the current stage.

The following section provides a comprehensive review of these themes.

### An Overview of Online Health Information Seeking Behavioral Research

Research on online health information-seeking behavior has been richly documented. From the scan of the current research, we can find that online health information-seeking behavior mainly focuses on the construction of online health information platforms, online health information-seeking behavior with different group characteristics, online health information content evaluation, and online health information user satisfaction. This section focuses on combing the research on online health information-seeking behavior from these perspectives.

#### Online Health Information-Seeking Platforms

Research and development of web-based health information access platforms/systems have been practiced in various countries, mainly in the United States, the United Kingdom, and China. The earliest one can be traced back to 1989 when the Center for Health Systems Experimentation and Analysis at the University of Wisconsin and several research institutes and hospitals collaborated to develop the Comprehensive Health Enhancement Support System (CHESS), a health information service website. CHESS is a health information service for people with life-threatening diseases such as cancer, leukemia, and HIV (Taylor et al., 1994). Information is provided by clinical experts and patients, and the main services include information query services, communication services, log services, and analytical services (Gustafson et al., 2005; Wang and Zhang, 2013). In 1998, the National Library of Medicine (NLM) and the American College of Physicians (ACP) Foundation partnered to develop MedlinePlus, a user-based health information service, to increase user awareness of web-based health information access and to help improve the quality of health information. MedlinePlus is a user-based health information service designed to raise awareness and help users understand online health information access (Miller et al., 2004). In 2002, to facilitate the public's access to quality health information resources, NLM relaunched Information Rx. The platform's primary role is to provide medical personnel and other professionals with the opportunity to communicate with patients while providing patients with information prescriptions and guiding patients to MedlinePlus's resources. That enhances the interaction between doctors and patients and effectively plays a role in health information literacy education (Thomas, 2010).

The UK's first online health information platform was Patient, launched in 1996, known for its high-quality health, lifestyle, disease, and other medical-related topics. It provides the public with up-to-date information on health-related topics through comprehensive presentations (readable online or in print), blogs, health advice, and videos. All information is edited and reviewed by physicians with years of medical expertise. Users can also visit the “Inquirer” function to self-diagnose any health conditions they may have (Patient, 2020). In 2004, the UK Health Service launched the Better Information, Better Choices, Better Health 3-year program to enhance health literacy training for healthcare professionals and facilitate communication between healthcare professionals and patients. A central component of the initiative was the deployment of digital health information and the development of health information standards to ensure the quality and accuracy of information content (Department of Health London., 2004). That has led to establishing several web-based health information services by local health services in the UK. For example, Patient Opinion is an open health information exchange platform created in 2005 in the UK. Health care workers or patients can share their treatment experiences and provide information to visitors/managers to help others get better services and provide advice and suggestions to managers (Chen et al., 2012).

China's health information search platform, which started in 2000 with the establishment of Ding-xiang-yuan, shares health information suitable for the general public and focuses on the exploration of professional and authoritative medical expertise. The platform covers daily health information, case studies, clinical drug use, network hospitals, hot research, academic research, medical education, and other aspects. It is an important platform connecting hospitals, doctors, researchers, patients, pharmaceutical enterprises, higher education, and other fields (Yan, 2012). In 2004, the platform was officially launched, and its functions integrate doctor-patient communication, drug data, network triage and consultation, health information search, etc. It covers the complete medical consultation process from online self-diagnosis before the consultation, online consultation, telephone consultation, drug inquiry and purchase, specialist registration to appointment booking, consultation guide, accompanying consultation during the consultation period to rehabilitation, follow-up consultation, and data tracking after the consultation, providing patients with convenient and fast services (Shuang et al., 2014). Although China's platform started a little later than other countries, thanks to the mature network application environment, China has made greater innovations and breakthroughs in feature richness, integration of medical resources, online medical applications, education and training, and industry expansion.

After years of development, with the advent of the mobile Internet era, mobile computing devices (such as smartphones and tablets) have greatly influenced many fields, including the medical health field. Online health information platforms have also been extended from traditional web-based platforms to mobile terminals with enhanced social networking attributes, gradually evolving toward mobile health services. In addition to web-based information search, mobile devices are equipped with a Global Positioning System (GPS), high-quality cameras, audio recorders, and just-in-time communications to meet the mobile needs of the healthcare environment (Ventola, 2014). Unlike the comprehensive health information on traditional web platforms, the positioning of mobile health information service platforms is clearly differentiated and roughly divided into three types.

##### Intelligent Mobile Clinic Platform

The most representative product of this type is Google Health, a personal health information service launched by Google in 2006. The service allows Google users to voluntarily enter their health records (such as their health status, drug use, and possible allergens, etc.) into the Google Health system, and through the analysis of this information, provide users with health care advice. In 2020, Google Health integrated DeepMind's Health business, making a breakthrough in the application of artificial intelligence (AI) technology in healthcare. Through intelligent calculation, analysis, and automatic reasoning of health data, it can assist clinical nurses, doctors, experts, and the public to make medical decisions, so as to improve the efficiency of diagnosis and treatment. Users can describe their physical condition to the platform at any time, communicate with smart customer service, upload video information, or upload biometric data using wearable devices. The AI technology can generate an assessment of an individual's health based on the analysis results, and give immediate advice on medical treatment, drug purchase, or treatment.

##### Mobile Service Platform for Medical Institutions

This type is primarily a way for healthcare organizations to implement mobile healthcare in the form of mobile applications, thereby easing clinical communication between providers and patients and improving hospital workflow. For example, AirStrip offers a mobile, interoperable platform that allows care coordination between multiple devices and multiple care settings. Data from an electronic health record, health information exchanges, medical devices, and other monitoring solutions can be accessed between smartphones, tablets, and computers from hospitals, post-acute care centers, and community-based care organizations. The AirStrip platform gives providers a tool to compact all this data into a single platform that can be accessed *via* telemedicine and integrated with systems from other vendors, enabling patients and physicians alike to gain real-time access and monitoring of complete medical procedures using mobile devices.

##### Cross-Platform Integration of Mobile Health Services

In China, mobile health information services have adopted a different approach. Thanks to the maturity and popularity of technologies such as social platforms, short videos, mobile payment, and big data analysis, a full-media delivery matrix based on the content of the original platform as a basic resource have been formed. Mobile health information service is no longer limited to a single professional app, but through information sharing among health information platforms, various types of health information are converged to high frequency used instant messengers and life service platforms, such as WeChat and Alipay. This not only integrates the scenario of health information acquisition into the daily life of the public, but also reduces the barriers to health information acquisition. The large amount of data on the social and lifestyle habits of users in these platforms has also facilitated the accurate delivery and utilization of health information. Especially in the recent COVID-19 dissemination chain tracking, it has played an important role.

Overall, based on the collation and sorting of the above-mentioned representative platforms, the development history of online health information platforms can be broadly summarized into eight basic features. First, the scope of information provided has been widened from a single medical field to the provision of comprehensive health information. Second, the type of service recipients has been expanding from a single type of patient service to include professionals, doctors, researchers, the public, and other types of people. Third, the degree of service specialization and functions are continuously strengthened from basic information inquiry service to comprehensive health information service covering lifestyle, self-diagnosis, health training, experience exchange, etc. Fourth, the types of health information are constantly enriched. From only logbook and case information, it has developed to a full range of health information services integrating image data, health briefings, network media, academic research, etc. Fifth, the form of interaction has been enhanced. From the initial communication between doctors and patients, it has developed into a multi-dimensional interaction including experts, doctors, patients, medical and nursing staff, researchers, and the public. Sixth, the platform information quality management continues to be strict. From relying only on the professionalism or experience of information providers, the platform has gradually established and improved health information standards, constantly optimized and standardized health information content, and ensured the quality and accuracy of information content. Seventh, the transmission path is continuously optimized. From web-based health information provision, it started to shift to intelligent, social, and shared health information provision based on mobile terminals. Eighth, information service technology continues to advance. From the early health information collection, distribution, and retrieval technology, it has developed to the realization of intelligent diagnosis and treatment technology based on big data analysis and integration of AI technology.

#### Characteristics of Online Health Information Seeking Groups

Throughout the research of information behavior, group characteristics have always been an important dimension to classify information-seeking behavior. A series of studies have shown that online health information-seeking behavior is highly correlated with the user's gender, age, race, health status, psychological factors, region, education level, economic status, and other group characteristics.

##### Gender Characteristics

Regarding gender, most studies show that women are more likely than men to search the Web for health information about themselves or others. Research by Mirowsky et al. suggests that women need this information more because they experience more chronic diseases, dysfunction, high morbidity, and psychological distress (Mirowsky and Ross, 1995). Second, Muller found that women are more interested in online health information because they are more likely than men to care about the health of their families (Muller, 1990). Drentea et al. (2008) also believe that women are more likely than men to search for information about specific medical issues, medication or processes, and depression, anxiety, stress, and mental health. Men, on the other hand, are more likely than women to question the veracity of health information online and to question health information based on multiple sources of information, with much of the focus on the diagnosis and treatment of the disease (Cotten and Gupta, 2004).

##### Age Characteristics

Regarding age, most research findings focus on the study population among adolescents, young adults, and older adults. Yu et al. (2009) conducted a questionnaire survey on 2,577 adolescents in Shanghai and found that the content and frequency of adolescents' search for health information *via* the Internet were related to gender, length of time spent online, mother's education level, average monthly allowance, and conscious academic performance. Teens with health risk behaviors used the Internet more than those without risk behaviors to search for information about related behaviors. Boys were more concerned about health information on violence, sexual health, smoking, drugs, and alcohol. Gowen (2013) used semi-structured focus groups to investigate how young people used the Internet to obtain information and support about their mental health. They use online information to challenge what they hear from their doctors or to help them prepare for doctor visits, searching for things like understanding treatment options, analyzing their mental health, and learning about the side effects of prescription medications. Compared to younger adults, older adults are less health literate online and are mostly “adopters” of traditional information. A study conducted by the Pew Research Center showed that more than half of older adults age 65 and older access the Internet, especially to learn more about personal health issues (Medlock et al., 2015). Manafo and Wong (2012) used a qualitative approach based on grounded theory to collect online health information-seeking behavior of community-based older adults aged 55–70 years through in-depth interviews and showed that older adults had higher trust in online health information and were more concerned with health information about diseases, medicine, health care reform, nutritional diets, and wellness care in terms of content.

##### Ethnic Characteristics

Different races are often the reason why people's online health-seeking behavior is different. Using data from the 2007 Health Tracker Household Survey, Rooks found significant differences between Hispanics and European Americans in searching for online health information and using it to communicate with doctors. European Americans are more concerned about their health, and the proportion of users searching for health information is much higher than in other races. European Americans pay the most attention to health information in terms of health information, followed by Asian-Americans and, finally, African-Americans and Hispanics. African-Americans and Hispanics are better at using searched information to improve their health among users who search for health information (Rooks et al., 2012).

##### Health Status

There is a strong correlation between individual health status and online health information searching behavior. The type and frequency of search were different for different health statuses. Hou Xiaoni et al. conducted a questionnaire survey among 400 outpatients in Beijing's tertiary hospitals to analyze the online health information content seeking of outpatients. It was found that the search content of patients with diseases mainly included etiology, treatment methods, medication, etc., with particular attention paid to evaluation information of hospitals and doctors (Hou and Sun, 2015). People with chronic diseases are more concerned about online health information to help them make treatment decisions and seek long-term interaction with medical professionals to focus on ways to improve various health conditions, such as diet safety, fitness programs, etc. (Fox, 2007). Basch used a questionnaire to investigate online health information resources among 443 patients with cancer at urban cancer centers. Studies have found that more than 40% of cancer patients often use the Internet to obtain health information related to cancer treatment. The most concerned content includes cancer prevention methods, drugs, side effects, clinical trials, pain management, and other cancer patients' experience sharing.

##### Psychological Characteristics

Most studies have shown that the search for online health information is related to mental health quality. Myrick surveyed nearly 697 college students from a public University in the southeastern United States and analyzed their psychological factors in seeking health information. It was found that there were a large number of suspected diseases among college students, and paranoia and anxiety drove them to seek health information on the Internet in order to reduce their anxiety (Myrick et al., 2016). Lagoe selected 245 adults from the US and investigated their online health information-seeking behavior's psychological factors, using structural equations to model relationships between personality traits, health anxiety, and online health information search. The study found that people's fear of health leads to obvious anxiety traits, and the degree of health anxiety is directly related to the frequency of their online-seeking behavior. This phenomenon is particularly pronounced in people with symptoms such as neuroticism, depression, and suspected illness, who cannot tolerate health uncertainty and are more likely to find information online and seek a sense of self-efficacy quickly. This feeling of practical experience greatly affects their health information-seeking behavior (Lagoe and Atkin, 2015).

##### Regional Characteristics

The residential area often affects the online health information-seeking habits of the residents in the area. Jo et al. (2010) conducted a telephone survey of 10,325 South Korean people living in Gangwon province and the metropolitan city of Incheon about their online health information seeking over the past year. The study found that people living in metropolitan cities use health information on the Internet at a higher rate than people living in smaller cities. In terms of search content, residents in metropolitan cities pay more attention to health information about health care. In comparison, residents in small cities pay more attention to information about drug efficacy. Wathen and Harris (2007) interviewed 40 women living in rural medically deficient counties in Ontario, southwestern Canada, and found that due to medical treatment inconvenience, they often searched the Internet for matters needing attention and treatment for daily maintenance of diseases. Also, due to the lack of medical services in the residential areas of African Americans in big cities of the United States, most of the residents here choose to seek treatment for diseases through the Internet to solve their health problems through physical means or medicine purchase (Morey, 2007).

##### Education Level

Several studies have shown that the level of education plays a key role in online health information seeking. Lorence and Park (2007) analyzed the relationship between health information seekers' educational characteristics, Internet technology, and Internet health information seeking and found that the popularization of technology could not effectively improve users' Internet health information seeking ability in groups with low education levels. Lim et al. (2011) found that Singaporean women with a University degree or higher were more likely to choose the Internet as the primary channel for seeking health information, mainly because these highly educated women had higher online information identification skills, a better understanding of health information, and higher acceptance of online health information platforms.

##### Economic Status

It is generally accepted that income levels determine the quality of health resources. However, in the area of online health information seeking, there are different views. Due to the lack of financial resources, some low-income families rely more on online health information platforms for family health management. Because of online platforms' sharing nature, low-income groups have equal access to online health information, eliminating the information gap and information inequality caused by information asymmetry (Zhao, 2009). Nangsangna and Vroom (2019) conducted questionnaires and interviews with patients in three local hospitals and used regression analysis to determine the factors influencing online health information seeking. The study found that computer ownership in the home was an important factor influencing the use of the Internet for health information seeking. Those with higher incomes had higher participation in owning a personal computer at home and were more likely to use the Web to find health information.

#### Online Health Information Quality Assessment

With the development of network technology, the platforms and channels for providing online health information are becoming more abundant. However, at the same time, the problems of information pollution and information overload also come along with it, especially the information involving public health, whose professionalism and accuracy are extremely important. Therefore, effective evaluation of online health information is an important way to filter undesirable online health information, improve the quality of health information, and protect users' life safety (Zhang et al., 2014). At present, the evaluation of online health information is mainly carried out by online health information evaluation tools.

##### Evaluation Tool

The research on the evaluation of online health information by Wilson and Risk (2002) and Zhang et al. (2003) has been collated in detail and divided into four types according to different evaluation methods.

- Third-party certification categories: such as HONcode (Medical Health Website Code of Conduct), Med CIRCLE (Online health information Assessment Program), URAC (Medical Website Certification Program), etc., mainly on the site content authority, security, editing methods, links, and other aspects of the norms. According to each certification standard, the health information website is evaluated by a third party. The website that complies with the evaluation specification can display the certification mark on the website.
- Code of Conduct: such as AMA (Medical and Health Information Website Guide), eHealth Code of Ethics (Electronic Health Ethics Code of Conduct), HI-Ethics (Medical Internet Code of Ethics), etc., mainly for health information-related website content, advertising, funding, privacy, security, and other aspects of the norms. Health information website operators can refer to the above specifications for self-management.
- User evaluation category: such as DISCERN (Disease treatment selection resource evaluation system), is mainly used to help users assess the quality of disease treatment information questionnaire system. Health Information Website content can be used as a tool to open user assessment or by the site manager to evaluate their own and improve the site's quality.
- Information filtering category: such as OMNI (organization of medical network information evaluation standards), the main assessment of health information website resources target users, authority, origin, charging, hardware requirements, copyright, etc., in the process of user search for health information, according to the above criteria for the results of information filtering to ensure the quality of search results.

##### Assessment Practices

Based on the above evaluation tools, scholars at home and abroad have carried out many evaluations on the online health information platform. Fast et al. (2013) used HONcode and Discern Plus tools to analyze the quality of more than 60 websites related to urology's online health information resources. They found that only 25% to 30% of the websites were certified by HONcode. In Discern Plus score, the evaluation of “whether the treatment will affect the quality of life,” “describe the risk of treatment,” and “the details of the information source” is the lowest score. Studies have shown that patients' and their parents' quality of information when making decisions and choosing treatment is very poor, and users are not informed of treatment risks. Patients and their parents cannot be correctly guided to make effective decisions. Park et al. (2005) took breast cancer, asthma, depression, obesity, and other diseases as the search targets for health information and selected the top 9 popular websites as the evaluation objects. The study found that the overall quality score of Internet health information in South Korea was 2.0, while that of commercial websites was 1.8. The overall score was lower than the hospital website's average score of 2.4. That means that the Internet health information in South Korea almost fails to meet the pundit standards, and the information quality has serious problems, which is in urgent need of quality improvement. Li et al. (2017) use pundit and JAMA tools to evaluate the credibility, detail, information disclosure, author, attribution, and update time of the contents of 17 websites that provide hypertension health information. Results show that there is only one website pundit rated as “excellent,” and none of them fully meet JAMA's evaluation criteria. Among them, 11 websites refer to false information, which shows the poor quality of online health information on hypertension.

#### User Satisfaction of Online Health Information

Although the use of online health information platforms is getting higher and higher, from the above research, it can be seen that the information quality problem has always existed, and different users of the online health information platform service recognition difference are also relatively large. In order to improve the quality of service of online health information platforms, scholars have carried out a lot of research on user satisfaction, collated them, and found that the factors affecting satisfaction mainly involve three aspects, namely, the authority of health information, the degree of interaction between health information platform and users, and the effectiveness of health information.

Ayantunde et al. (2010) conducted a 2-week questionnaire survey of patients and accompanying adults over 18 in the outpatient clinic of Nottingham City Hospital. It was found that the authority and trustworthiness of health information were the reasons why most of the respondents were satisfied with online health information. At the same time, those who had no experience searching online were strongly interested in searching if the health information was authoritatively verified, which shows that the authority of information is a prerequisite to attract users to use online health information platforms. Nsupa (2010) examined the comparison of the satisfaction of 178 patients with cancer with the online health information platform and their oncologists from the perspectives of both use and satisfaction theory (U&G) and media dependence theory (MSD). The study found that most patients viewed the online counseling platform as a better source of information than a care provider. They cared more about the level of empathy and time spent with the patient. This study emphasizes that effective interaction between online health platforms and users is an important factor contributing to user satisfaction. At the same time, Kim and Chang (2007) used the Technology Acceptance Model (TAM) to analyze users' acceptance of online health information platforms in terms of perceived ease of use (PEOU) and perceived usefulness (PU), as determined by the key features of “help with use” and “customized services Users” attitudes toward PEOU and PU. The results of the study revealed that little direct effect of PEOU was observed on the “help to use” function, while PU was more significant. This suggests that users are more interested in online health information platforms' effectiveness and do not have significant requirements for their ease of use.

### Modeling Online Health Information-Seeking Behavior

The above content provides a detailed analysis of various aspects and important elements of online health information-seeking behavior, which are important elements to guide the construction of online health information-seeking models. A well-developed model of online health information-seeking behavior can help healthcare professionals, patients, consumers, and researchers better understand their health information needs, grasp the correct communication methods, clarify appropriate seeking channels, standardize information-seeking behavior, and guide the research process. Previously, Freimuth and Longo have developed different health information-seeking models in their respective studies, which are described below.

#### Freimuth's Health Information Collection Model

**Figure 5:**
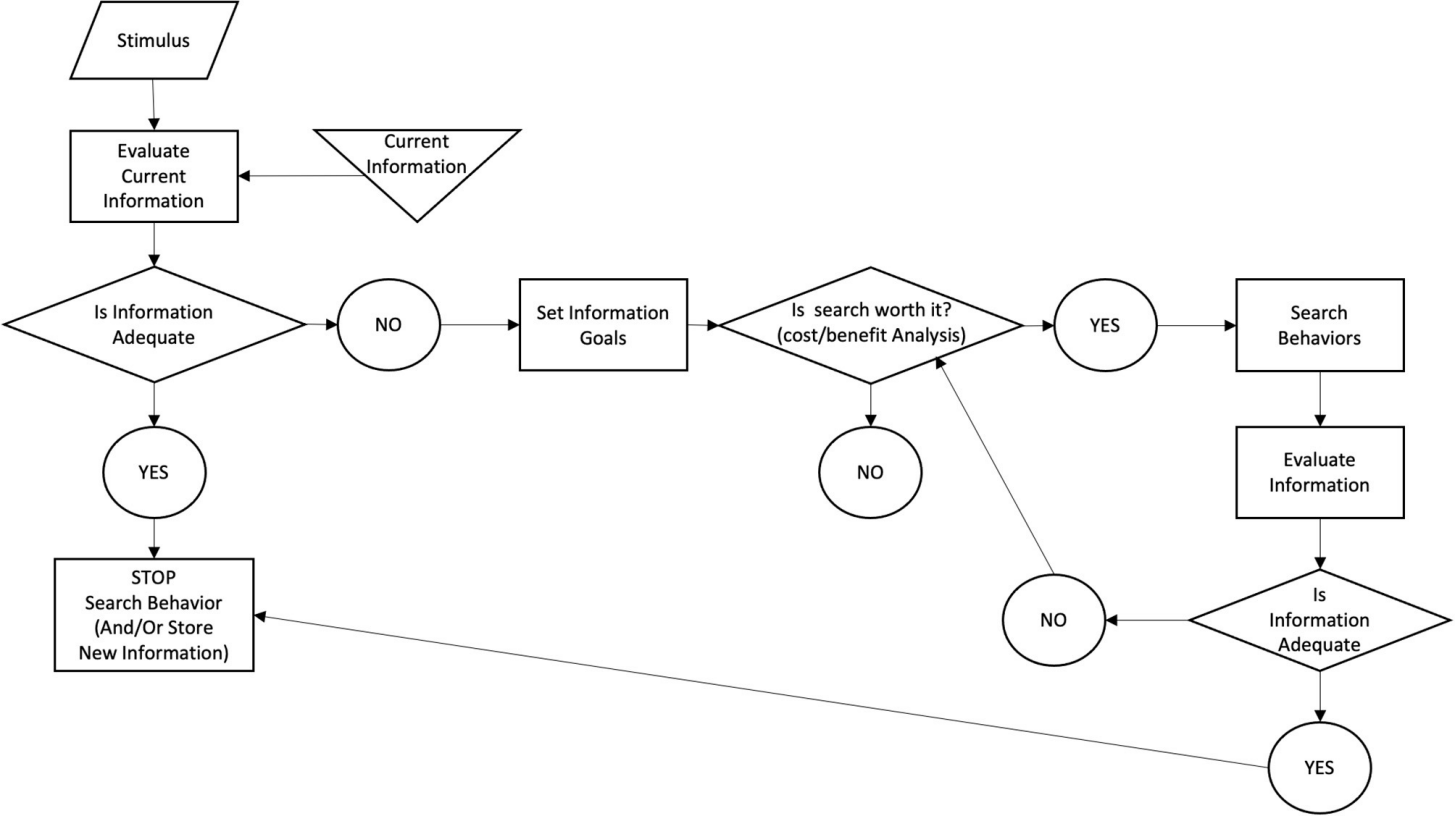
Freimuth's health information collection model.

Freimuth and Stein (1989) analyzed data from more than 1 million calls from patients with cancer recorded in the HISB Database [Cancer Information Service (CIS)] over the past 4 years, as well as a follow-up survey of more than 7,500 callers. This summarizes the general context in which people search for health information about various diseases and other health issues. Figure 5 provides a theoretical overview of how people search for health information, which shows that users generate a need for health information in response to external stimuli, obtain useful information to satisfy the need from the currently available health information database, and evaluate whether the current information is sufficient to satisfy the need. If it is satisfied, then the search ends, and if it is not satisfied, then the health information search goal needs to be redefined. The user weighs the cost-effectiveness of continuing the search. If it is worth searching, the search will continue to be triggered until sufficient health information is available to address the need.

#### Longo's Extended Model of Health Information-Seeking Behavior

**Figure 6:**
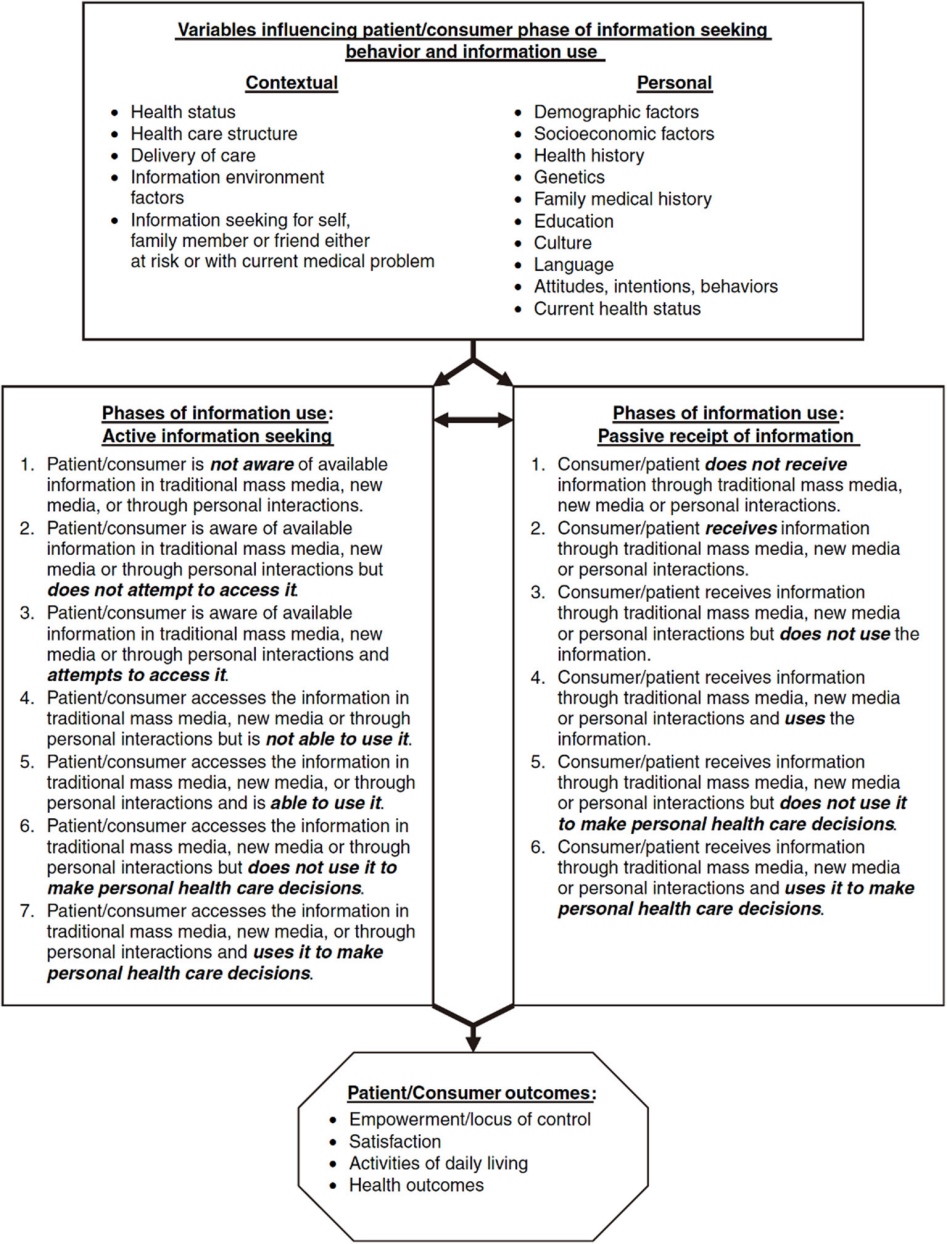
Longo's extended model of health information seeking behavior.

Longo (2010) surveyed a total of 121 women previously diagnosed with breast cancer in the Columbia and Central Missouri Kansas City areas and Newark, New Jersey, using a focus group approach to understand the medical status and health information use of these women. The survey found that many women did not intentionally seek health information, but that this health information could be used and have far-reaching effects, thus requiring consideration of the existence, role, and importance of “passive” information receipt vs. “active” information seeking; some respondents received health information from traditional print media rather than “new media” such as the Internet, which emphasizes the need for a comprehensive understanding of health information sources. In addition, personal and contextual variables also play a role in health information search. The authors suggest that information search results from complex interactions between multiple variables, each with different abilities to aid information use. The system in Figure 6 reflects Longo's extended model of health information-seeking behavior based on the above findings. The model illustrates that the patient/consumer's health information-seeking process may result in active or passive acceptance of health information, use decisions by judging the usefulness of the information content, and ultimately the impact of personal status, depending on the individual's status and context.

#### Online Health Information-Seeking Behavior Modeling

Combined with the discussion above and the comparison of the above models, it can be seen that both Freimuth's health information collection model and Longo's health information-seeking behavior expansion model are suspected to be too one-sided. On the one hand, Freimuth emphasizes the action of health information seeking and ignores the various internal and external factors that affect it. On the other hand, Longo lays too much emphasis on the various channels of receiving health information and their influence on the users' decision to use information under multiple variables of individuals and situations while neglecting seeking health information. Especially in the network environment, the above models can no longer meet the requirements of reflecting current health information-seeking behavior's characteristics and status and need to be supplemented and improved.

**Figure 7:**
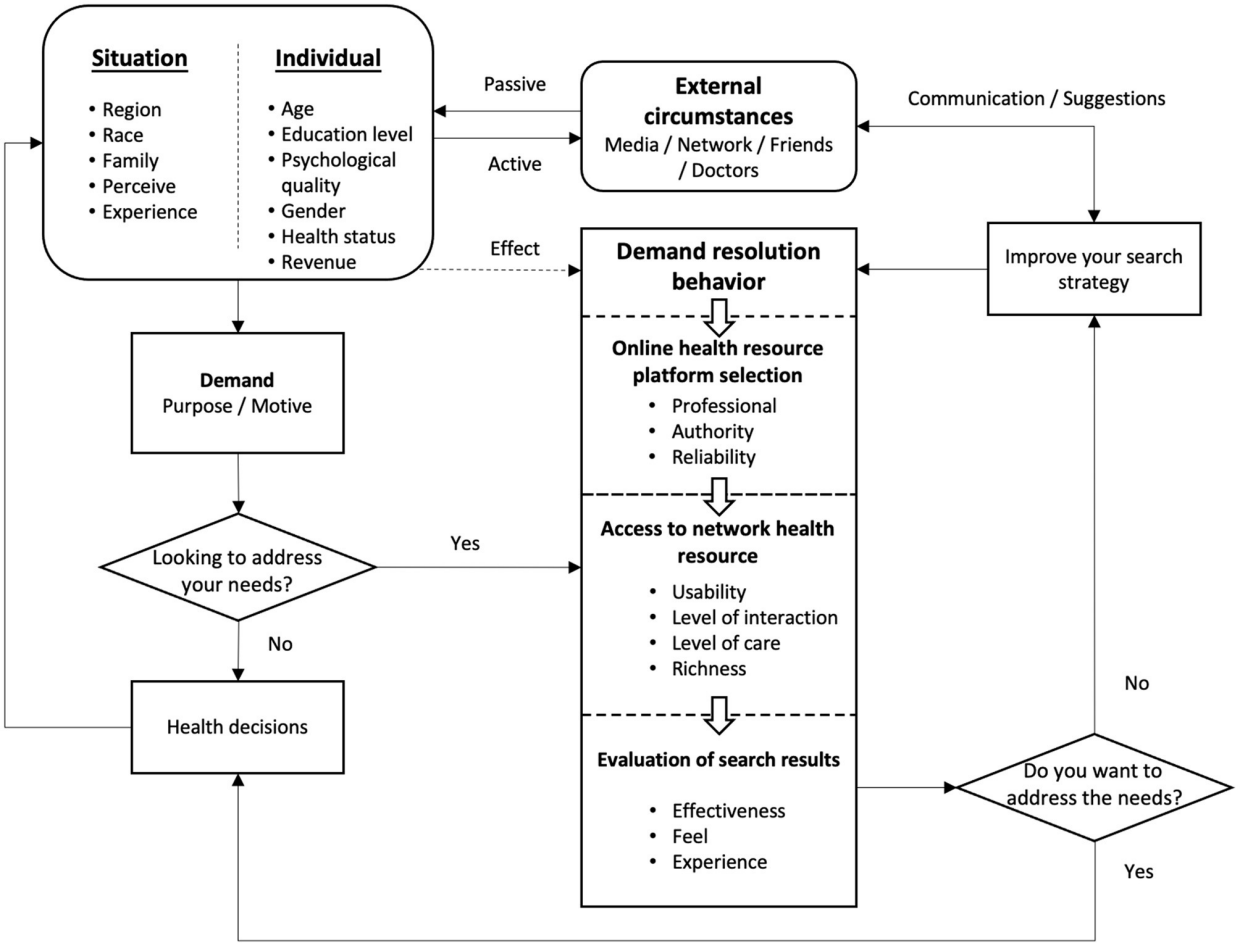
Online health information seeking behavior model.

This study combines the above model with the review mentioned above of online health information platforms, groups, contents, and satisfaction. It constructs a model of online health information-seeking behavior, as shown in Figure 7. The model starts from the active and passive influencing factors of individuals, situations, and external environment, and then generates the motivation and purpose of health information seeking, forms the demand of health information seeking, and triggers a series of demand-solving behaviors (online health information platform selection, online health resource acquisition, and outcome evaluation). The situation and personal factors have a certain influence on the searching behavior in this process. Finally, according to the search results to solve the level of demand, determine whether the further search and the external environment in the process of dialogue and exchanges will also affect the user further medical information search strategy, and trigger the solution behavior again until the health information needs are met, and to help users supporting health decisions, and the influence on the formation situation and personal.

## Discussion

In this review of research on online health information-seeking behavior, we have identified a number of researchers who have conducted relevant research from different perspectives and in different ways, and the articles listed in this paper are among the most widely cited and representative.

In the current state of research, the development is highly uneven in terms of the number of studies conducted across countries. Although the number of researchers in each country may limit the development of the field, health information is equally important to the public, especially in the state of an outbreak, and the quality and abundance of online health information may directly contribute to the effectiveness of the country's epidemic preparedness. Current research coverage and types, although already relatively abundant, focus on research on technology, information quality, and patients, while research on general users, influencing factors, future trends, and quality assessment is weak. Especially in the mobile Internet environment, there is still a lack of research on the construction, development, and evaluation of mobile platforms, and relevant influencing factors need to be broadened. Factors such as health information access devices, dissemination channels, and service tracking can be added to shift the focus of research and pay attention to new changes in the mobile environment. Although emerging research themes have gradually emerged in terms of the richness of research topics, the depth and persistence of research are not sufficient in terms of the period of emergence, and it is not easy to form systematic and mature research results. In addition, from the current research, the current certification in online health information platforms is not strong enough, the quality of the information in a large number of platforms is worrying, and there is a lack of relevant research from the perspective of government management.

## Conclusions and Suggestions

In the context of COVID-19, online health information has become one of the most important information needs of the public, and even the only channel for health information seeking in a specific period. At this time, the perfect and rational use of online health information platforms can help promote the prevention and control of global epidemics. In summary, this study systematically composes the research on online health information-seeking behavior. Overall, the current problems concerning the construction of online health information platforms are prominent, mainly reflected in the generally low quality of content, the bottom of accuracy, lack of perfect platform functions, and lack of mobile service platforms, which cannot meet the needs of different types of users. As a provider of online health information, it should simplify the search threshold of the platform, reduce the search cost of users, improve the efficiency of search feedback, guide users to use it, and increase the care and interaction of the platform for users. On the government side, reasonable health information management policies should be introduced to promote the benign development of online health information platforms and guarantee the safety and reliability of health information access. The model of online health information-seeking behavior proposed in this study will also help the implementation of the above improvement suggestions. It also constructs a complete topic mapping for researchers in this field.

## Limitations

Although this study attempted to systematically review research on online health information-seeking behavior and tried to propose a new research model. However, there are still some current research limitations that cannot be addressed. The limitations that exist are described below for future researchers to judge and improve.

- The WOS database was selected as the only data source for this study, and although it already covers most of the high-quality papers, it cannot cover the complete range of articles.
- This study utilized the Citespace tool to draw keyword co-occurrence mapping, and in the analysis of keyword co-occurrence, authoritative control of keywords was done manually, but subjective judgments due to human factors could not be excluded; in addition, there were inevitable omissions in the automatic analysis and mining of texts by the tool.
- This study constructs a new model of online health information-seeking behavior, but no empirical study has been conducted, which is a direction that can be studied in the future.

## Author Contributions

Z-WL completed the conceptualization, article writing, data collection, data processing, revision, and submission of this study.

## Conflict of Interest

The author declares that the research was conducted in the absence of any commercial or financial relationships that could be construed as a potential conflict of interest.

## Publisher's Note

All claims expressed in this article are solely those of the authors and do not necessarily represent those of their affiliated organizations, or those of the publisher, the editors and the reviewers. Any product that may be evaluated in this article, or claim that may be made by its manufacturer, is not guaranteed or endorsed by the publisher.

## References

[B1] Ayantunde A. A. Welch N. T. Parsons S. L. (2010). A survey of patient satisfaction and use of the Internet for health information. Int. J. Clin. Pract. 61, 458–462. 10.1111/j.1742-1241.2006.01094.x 17313614

[B2] Chen Q. Song S. Zhao Y. (2020). Impact of information overload on user information avoidance behavior in public health emergencies: an empirical study based on the COVID-19 information epidemic. Libr. Inform. Serv. 41, 76–88. 10.12154/j.qbzlgz.2020.03.011

[B3] Chen X. Xin S. Zhang S. (2012). Patient Opinion Website's Implications for China's Doctor-Patient Interactive Website Construction. Hospital Management in China.

[B4] Cotten S. R. Gupta S. S. (2004). Characteristics of online and offline health information seekers and factors that discriminate between them. Soc. Sci. Med. 59, 1795–1806. 10.1016/j.socscimed.2004.02.020 15312915

[B5] Department of Health London. (2004). Better Information, Better Choices, Better Health: Putting Information at the Centre of Health. Department of Health London.

[B6] Drentea P. Goldner M. Cotten S. Hale T. (2008). The association among gender, computer use and online health searching, and mental health. Inform. Commun. Soc. 11, 509–525. 10.1080/13691180801999019

[B7] Fast A. M. Deibert C. M. Hruby G. W. Glassberg K. I. (2013). Evaluating the quality of Internet health resources in pediatric urology. J. Pediatr. Urol. 9, 151–156. 10.1016/j.jpurol.2012.01.004 22281281

[B8] Fletcher R. Radcliffe D. Levy D. Nielsen R. K. Newman N. (2015). Reuters Institute digital news report 2015: supplementary report. Digital News Report 2015, Reuters Institute for the Study of Journalism.

[B9] Fox S. (2007). E-*Patients* with a *Disability* or *Chronic Disease*. Pew Internet & American Life Project. Washington, DC: PEW.

[B10] Freimuth V. S. Stein J. A. (1989). Searching for Health Information. Philadelphia, Pa: University of Pennsylvania Press. 10.9783/9781512816075

[B11] Gowen L. K. (2013). Online mental health information seeking in young adults with mental health challenges. J. Technol. Hum. Serv. 31, 97–111. 10.1080/15228835.2013.765533

[B12] Gustafson D. H. Hawkins R. P. Boberg E. W. McTavish F. Owens B. Wise M.. (2005). CHESS: 10 years of research and development in consumer health informatics for broad populations, including the underserved, in: Consumer Health Informatics. Health Informatics, eds Lewis D. Eysenbach G. Kukafka R. Stavri P. Z. Jimison H. B.. (New York, NY: Springer), 239–247. 10.1007/0-387-27652-1_20

[B13] Hou X. N. Sun J. (2015). A study on internet health information seeking behavior of outpatients in Beijing's tertiary hospitals. Libr. Intell. Work 59, 126–131, 111. 10.13266/j.issn.0252-3116.2015.20.021

[B14] IResearch (2020). Epidemic Watch - 2020 China Information Flow Industry Development Change Inventory. Available online at: https://report.iresearch.cn/report/202006/3589.shtml?s=enable (accessed March 15, 2021).

[B15] Jo H. S. Hwang M.-S. Lee H. (2010). Market segmentation of health information use on the Internet in Korea. Int. J. Med. Inform. 79, 707–715. 10.1016/j.ijmedinf.2010.07.006 20810307

[B16] Kim D. Chang H. (2007). Key functional characteristics in designing and operating health information websites for user satisfaction: an application of the extended technology acceptance model. Int. J. Med. Inform. 76, 790–800. 10.1016/j.ijmedinf.2006.09.001 17049917

[B17] Lagoe C. Atkin D. (2015). Health anxiety in the digital age: an exploration of psychological determinants of online health information seeking. Comput. Human Behav. 52, 484–491. 10.1016/j.chb.2015.06.003

[B18] Li P. Tang G. Yang H. Du Z. (2017). Quality evaluation of health information on hypertension in a ubiquitous web environment. Mod. Prevent. Med. 20, 85–88.

[B19] Li S. Wu Y. Zhang F. Xu Q. Zhou A. (2020). Self-affirmation buffers anxiety reactions to the new crown outbreak: a randomized controlled study. Psychol. News 52, 886–894. 10.3724/SP.J.1041.2020.00886

[B20] Lim S. Xue L. Yen C. C. Chang L. Chan H. C. Tai B. C.. (2011). A study on Singaporean women's acceptance of using mobile phones to seek health information. Int. J. Med. Inform. 80, e189–e202. 10.1016/j.ijmedinf.2011.08.007 21956003

[B21] Longo D. R. (2010). Understanding health information, communication, and information seeking of patients and consumers: a comprehensive and integrated model. Health Expect. 8, 189–194. 10.1111/j.1369-7625.2005.00339.x 16098149 PMC5060298

[B22] Lorence D. Park H. (2007). Study of education disparities and health information seeking behavior. Cyberpsychol. Behav. 10, 149–151. 10.1089/cpb.2006.9977 17305464

[B23] Manafo E. Wong S. (2012). Exploring older adults' health information seeking behaviors. J. Nutr. Educ. Behav. 44, 85–89. 10.1016/j.jneb.2011.05.018 22101129

[B24] Medlock S. Eslami S. Askari M. Arts D. L. Sent D. De Rooij S. E.. (2015). Health information–seeking behavior of seniors who use the internet: a survey. J. Med. Internet Res. 17, e10. 10.2196/jmir.3749 25574815 PMC4296102

[B25] Miller N. Tyler R. J. Backus J. E. (2004). MedlinePlus®: The National Library of Medicine® Brings Quality Information to Health Consumers, in Library Trends. Vol. 53., ed Mays T. L. (Bethesda, MD: NIH/NLM), 375–388.

[B26] Mirowsky J. Ross C. E. (1995). Sex differences in distress: real or artifact? Am. Sociol. Rev. 60, 449–468. 10.2307/2096424

[B27] Morey O. (2007). Health information ties: preliminary findings on the health information seeking behaviour of an African-American community. Inform. Res. 12, 297. Available online at: http://InformationR.net/ir/12-2/paper297.html (accessed March 15, 2021).

[B28] Muller C. (1990). Health Care and Gender. New York, NY: Russell Sage Foundation.

[B29] Myrick J. G. Willoughby J. F. Verghese R. S. (2016). How and why young adults do and do not search for health information: cognitive and affective factors. Health Educ. J. 75, 208–219. 10.1177/0017896915571764

[B30] Nangsangna R. D. Vroom F. D.-C. (2019). Factors influencing online health information seeking behaviour among patients in Kwahu West Municipal, Nkawkaw, Ghana. Online J. Public Health Inform. 11, e13. 10.5210/ojphi.v11i2.10141 31632607 PMC6788904

[B31] Nsupa T. (2010). The role of patient satisfaction in online health information seeking. J. Health Commun. 15, 3–17. 10.1080/10810730903465491 20390974

[B32] Park J. H. Jo B. R. Kim Y. I. Sin Y. S. Kim Y. (2005). Assessing the quality of internet health information using DISCERN. Lakartidningen 47, 137–139. 10.4258/jksmi.2005.11.3.235

[B33] Patient (2020). Health Information You Can Trust. Available online at: https://patient.info/about-us (accessed March 15, 2021).

[B34] Rooks R. N. Wiltshire J. C. Elder K. BeLue R. Gary L. C. (2012). Health information seeking and use outside of the medical encounter: is it associated with race and ethnicity? Soc. Sci. Med. 74, 176–184. 10.1016/j.socscimed.2011.09.040 22154611

[B35] Shuang S. Liu F. Yuting Y. Zhonghua Y. (2014). Analysis of the current situation of online medical information service platforms in China - 39health.com, seekmedicine.com and good doctor online as examples. Mod. Trade Indus. 26, 162–164. 10.3969/j.issn.1672-3198.2014.07.085

[B36] Taylor J. O. Gustafson D. H. Hawkins R. Pingree S. McTavish F. Wise M.. (1994). The comprehensive health enhancement support system. Qual. Manag. Health Care 2, 36–43. 10.1097/00019514-199402040-00007 10137606

[B37] Thomas M. (2010). The information rx program requires more promotion, more support and some adjustment. Evid. Based Libr. Inf. Pract. 5, 102–104. 10.18438/B8XW50

[B38] Ventola C. L. (2014). Mobile devices and apps for health care professionals: uses and benefits. P. T. 39, 356–364. 24883008 PMC4029126

[B39] Wang Y. Zhang S. (2013). Introduction and insights from CHESS and MedlinePlus, U.S. health information service websites. China Health Educ. 29, 852–854.

[B40] Wathen C. N. Harris R. M. (2007). “I try to take care of it myself.” How rural women search for health information. Qual. Health Res. 17, 639–651. 10.1177/1049732307301236 17478646

[B41] Wilson P. Risk A. (2002). How to find the good and avoid the bad or ugly: a short guide to tools for rating quality of health information on the internet. Commentary: On the way to quality. BMJ 324, 598–602. 10.1136/bmj.324.7337.598 11884329 PMC1122517

[B42] Yan L. (2012). Ding Xiang Yuan: The era of medical APP. IT Manager World 5, 2.

[B43] Yu C. Huijing S. Piye Z. Aihua A. (2009). Adolescent Online Health Information Seeking Behaviors and Their Correlation with Health Risk Behaviors. School Health in China. 30, 3.

[B44] Zhang F. Xu P. Liu Y. (2003). Progress of Foreign Medical Information Evaluation on the Internet. Medical information work. 24, 477–480. 10.3969/j.issn.1673-6036.2003.06.033

[B45] Zhang H. Ma J. Di J. (2014). A review of research on quality assessment of online health information. J. Med. Inform. 3, 6. 10.3969/j.issn.1673-6036.2014.03.001

[B46] Zhao S. (2009). Parental education and children's online health information seeking: beyond the digital divide debate. Soc. Sci. Med. 69, 1501–1505. 10.1016/j.socscimed.2009.08.039 19765874

